# Improving Medical and Surgical Care: A Qualitative Study of Medical Students’ Experience With Language Barriers in Healthcare

**DOI:** 10.7759/cureus.66505

**Published:** 2024-08-09

**Authors:** Mehrab Durrani

**Affiliations:** 1 Hospital Medicine, Royal Lancaster Infirmary, Lancaster, GBR

**Keywords:** medical education and learning classroom integrated, language impairment, language research, language barrier, psycho-behavioral, radiology reporting

## Abstract

Objective

This study aims to investigate the experiences of medical students in teaching and practice concerning communication with patients exhibiting limited English proficiency (LEP). With a growing population in the United Kingdom facing language barriers, this research explores the challenges encountered by medical students, emphasizing the need for enhanced medical education in this domain.

Methods

A qualitative technique was used to recruit 20 medical students enrolled in a Medicine MBBS degree program at a UK institution. Convenience sampling was utilized, and five separate focus groups were conducted. Semi-structured interview schedules guided discussions surrounding encounters with patients necessitating the use of interpreters. Thematic analysis was employed to identify recurring patterns and themes.

Results

Three primary themes emerged from the data analysis: interpersonal relations, feasibility, and duties and responsibilities. The findings underscore the challenges faced by medical students when communicating with patients having LEP. The study provides valuable insights into the nuanced aspects of these encounters, shedding light on the complex interplay between language barriers and effective healthcare delivery.

Conclusion

This research contributes to the limited body of qualitative literature on medical students' experiences in managing language barriers with patients. The identified themes provide a foundation for modifying medical education curricula to better equip students to address communication challenges associated with linguistic diversity. The study advocates for the integration of these experiences into medical training, emphasizing the broader implications for healthcare delivery and the reduction of health inequalities in linguistically diverse patient populations. The study also recommends the usage of up-to-date translation services built into the electronic healthcare records. This relationship minimizes inaccuracy in presenting the results of patient diagnostic films and tests, reduces wait times for treatment, and contributes to an overall cost reduction within this healthcare sector.

## Introduction

According to the latest UK census, around 2% (863,000) of the population of England and Wales had limited English proficiency (LEP) [1]. These individuals may experience challenges in communicating or understanding English, potentially hindering their societal integration. Notably, only 65% of individuals with LEP reported having “good health,” compared to nearly 80% of those with English proficiency [1]. While the proportion of individuals reporting “good health” decreases with age regardless of language skills [2], the data suggest significant health disparities that may be partially attributed to limited English competence.

LEP poses significant challenges in accessing healthcare and achieving desired outcomes from consultations. Extrapolating from reference data, Gill et al. estimated that nearly 7.3 million general practice (GP) appointments annually may require interpretation services [3]. This highlights the need for practical training for clinicians to effectively work with both interpreters and patients. Such training is crucial for optimal clinical outcomes and will become increasingly necessary in an era of increased immigration and multi-ethnic populations.

Interpretation services

Various interpretation services are available to those with LEP. To address inequalities in care, the National Health Service (NHS) employs third-party providers to supply interpreters for consultations. However, variations in training pathways across companies may lead to inconsistencies in interpreter competency. The Royal College of Physicians outlines two primary service categories: written translation (e.g., brochures, therapy guidance, letters) and oral interpretation (face-to-face, phone, or video) [4]. While each service has its advantages, challenges such as arrangement difficulties, accessibility, and patient comfort can hinder effective communication [5]. This may result in the use of informal communication methods that may not yield optimal health outcomes [5].

Family members have also been used as translators in consultations. Despite established trust, this practice carries its own cautions. Family members may filter information based on their perceptions of the patient's intelligence or psychological resilience, potentially excluding important details [6]. Furthermore, researchers have found that family members may withhold information based on their views of the patient's best interests [6].

Interpretation services and clinical outcomes

The relationship between interpreters and positive clinical outcomes is well-established. A systematic review by Karliner et al. in 2007 found that using professional interpreters in LEP patients correlated with improved care outcomes for patients with LEP, including improved clinical outcomes, patient understanding, and satisfaction [7]. However, most reviewed studies were conducted in the US with Spanish-speaking patients, potentially limiting generalizability to other countries and languages [7]. Notably, the UK's primary non-native languages include Polish, Punjabi, Urdu, Bengali, Gujarati, and Arabic [1]. While UK-based studies are scarce, findings from other countries may hold implications.

Another systematic review by Flores in 2005 concluded that optimal communication, patient satisfaction, and fewer translation errors arise when interpreters are involved in care [8]. However, limitations exist in the study such as failing to distinguish between interpreter types (professional, untrained staff, or relative), potentially impacting patient experiences and care quality [8]. These methodological problems pose a large question on the study’s validity and accuracy. An aspect that was not included in both review studies was the cost-to-benefit ratio of using an interpreter. Having this would significantly reinforce that interpreters could likely ultimately save the NHS a lot of expenditure. Advantages of interpreters include enhanced clinician-patient communication, allowing patients to experience fewer delays in diagnosis, and therefore quicker treatment commencement improved adherence, which in turn means reduced hospital time and thus less money spent on unnecessary or incorrect procedures [8,9]. With patients consistently showing greater satisfaction with interpreters being involved, and clinical outcomes being improved too, doctors and other healthcare staff must have the correct training to work effectively and productively with interpreters.

Communication training for medical students

Given the positive impact of interpreters on patient satisfaction and clinical outcomes, doctors and healthcare staff must receive training in working effectively with them. However, this type of education is often overlooked or only briefly covered in non-clinical years. The few studies addressing medical student training with interpreters found significant knowledge improvements after intervention [10-12], highlighting the need for education in this area.

Quantitative studies, however, often lack opportunities for students to express feelings, perceptions, and emotions regarding LEP patient communication training. Understanding medical student experiences and behaviors can provide arguably more valuable context for quantitative data and inform more effective teaching tools.

Objective of the study

The Medicine MBBS degree is structured into two components. The first is non-clinical theory-based studies. This comprises the first two years of medical school. Next is practical education. Lasting from the third year up to the final fifth year.

It is of utmost importance for our future clinicians to be able to communicate with their patients effectively. It is essential to identify student reflections and perceived needs on their current LEP communication teaching, to ultimately create the most effective teaching tool. Therefore, the objective of this study will be to assess the medical student experience in teaching and practice regarding communicating with LEP patients. Investigating this is crucial to see whether medical schools are doing enough in their students’ perspectives to prepare them to be able to face the wide diversity of patients in the UK and reduce health inequalities through addressing the complex medical, ethical, and cultural challenges that may come with these patients.

## Materials and methods

Design

A qualitative approach with thematic analysis of focus groups involving medical students was deemed the most suitable method for studying the research aims. This naturalistic approach focuses on gathering, analyzing, and interpreting personal perceptions and opinions of individuals regarding specific phenomena [13]. A qualitative study was chosen due to its ability to delve deeper into the meanings, beliefs, attitudes, and behaviors underlying medical students' experiences with interpretation services. Quantitative methods, with their structured and numerically based approach, lack the flexibility to capture the emotional complexity embedded within qualitative data.

Participants

Convenience sampling with a snowballing component was employed for participant recruitment. The inclusion criterion was being a UK medical student at any stage of their degree. Students in non-clinical years were excluded due to limited exposure to clinical settings and their inability to adequately respond to the research questions. Snowballing involved identifying eligible students and recommending their colleagues who might also be interested in participating. Approximately 100 students were invited to participate in the study, this represented approximately one-quarter of the cohort of medical students available in the clinical years of their study. Thirteen students were interested and aided in recruiting a further seven via snowballing.

Medical students were invited via email, accompanied by an invitation letter (Table 2: Appendix 1), a consent form (Table 3: Appendix 2), and an information sheet detailing the study purpose, procedures, and ethical considerations (Table 4: Appendix 3). Students from diverse backgrounds were invited to ensure the captured perspectives reflect the experiences of a varied group of individuals with differing language proficiencies and educational backgrounds regarding patient communication.

Twenty students were recruited and divided into focus groups of three to six individuals each. Utilizing snowballing allowed grouping based on prior familiarity, fostering a sense of security within the groups and encouraging open information sharing. Participants' multilingual abilities were also recorded, approximately 8 languages were spoken with over 10 distinct dialects. Participants were aged between 20 and 27 years old. This structure provided ample opportunity for individual expression and facilitated efficient data analysis and coding. Participant information is further detailed in Table 1.

**Table 1:** Participant demographics

Focus Group	Participant number	Stage of study	Sex	Multilingual ability
1	1	Stage 5	Male	Bilingual
	2	Stage 5	Male	Bilingual
	3	Stage 5	Male	Bilingual
2	4	Stage 4	Male	Bilingual
	5	Stage 4	Male	Bilingual
	6	Stage 4	Male	Bilingual
	7	Stage 4	Male	Monolingual
3	8	Stage 5	Male	Bilingual
	9	Stage 4	Female	Monolingual
	10	Stage 4	Female	Bilingual
	11	Stage 4	Female	Bilingual
4	12	Stage 3	Male	Bilingual
	13	Stage 3	Male	Bilingual
	14	Stage 3	Male	Monolingual
	15	Stage 3	Male	Bilingual
	16	Stage 3	Male	Bilingual
	17	Stage 3	Male	Monolingual
5	18	Stage 5	Female	Bilingual
	19	Stage 5	Male	Monolingual
	20	Stage 4	Male	Bilingual

Materials 

Focus group discussion topics included thoughts surrounding current teaching and the experiences and challenges medical students encounter working with LEP patients. Further discussion revolved around suggestions for improving training for medical students. Having a semi-structured design allowed for the interviewer to cover a series of open-ended topics but also the freedom to allow the discussion to follow a natural development, demonstrating an empathetic approach and also allowing for information that had not been anticipated to be shared. The interviewer adopted a facilitative stance and used prompts in order to gain more detail and further explore particular subjects, or probed others to allow them to get directly involved in the discussion and to delve deeper into the student’s feelings. Prompts were catered specifically for the medical student participants to allow for more data to be acquired for analysis. After the focus groups were completed, participants were sent a debrief sheet (Table 6: Appendix 5).

Procedure 

Due to the restrictive nature of the COVID-19 pandemic, the interviews were held via the video conference platform Zoom. After giving informed consent, participants were invited to attend a focus group on Zoom run by the lead researcher. Each group call aimed to last at least 45 minutes but was made to be flexible enough to carry on for longer if needed. This was to give sufficient time to allow each individual to become comfortable enough to answer questions. The sessions lasted 20 minutes as the shortest, with 55 minutes being the longest. An advantage of using Zoom is that since everything is recorded, the program automatically produces a transcript, which was inspected multiple times for any discrepancies.

Data analysis 

Once the data were transcribed, it was then coded, analyzed, and interpreted by the inductive thematic analysis framework [14]. Meaning, themes, and patterns that arose within the discussion were reported and scrutinized [15]. In terms of the analysis approach, this study was open to developing both semantic and interpretative themes, which primarily focus on the data at a surface level as well as interpretation and theories of the patterns and meanings behind the participants’ answers [16].

Researcher

The researcher who conducted the focus groups and analyzed the data is a Clinical Psychology student, currently intercalating an MSc from Medicine MBBS. Therefore, it can be said that preconceptions regarding the topic may have been held from the researcher’s time spent studying medicine and may have a bearing on the involvement with the topic. However, every effort to stay neutral was implemented to ensure the findings were objective and reliable. These included the use of external reviews from supervisors to review study design, data collection, and analysis processes. Also training on recognizing and minimizing personal biases during the research process such as using a reflexivity journal where the researcher regularly recorded their thoughts and potential biases throughout the research process, promoting self-awareness and transparency.

Ethics

Full ethical approval was received from the Newcastle University Faculty of Medical Sciences Ethics Committee (Newcastle University Research Office, Research Policy, Intelligence, and Ethics Team. Reference Number: 1108/2020). Prior to all focus group interviews, information sheets describing the rationale and contents of the study, as well as consent forms containing confidentiality procedures and right-to-withdraw measures were given to every participant. These were required to be read and signed before the medical student would be classed as eligible for the study. As the focus groups were taking place via Zoom, a number of ethical dilemmas had to be addressed before allowing the study to go ahead. Firstly, video recordings of the group were taking place, therefore informed consent from the student participants was obtained, and once transcribed, the video recordings were subsequently deleted. Another ethical dilemma was the storing and transcribing of data, some video conference platforms both store and transcribe their data through the use of third-party companies. However, Zoom has its own cloud storage service and also conducts its own transcripts, thus this dilemma was averted. In addition to this, once the transcribed data had been manually proofread and checked for accuracy, participant names were replaced with a designated number to guarantee anonymity to readers of this study.

## Results

**Figure 1:**
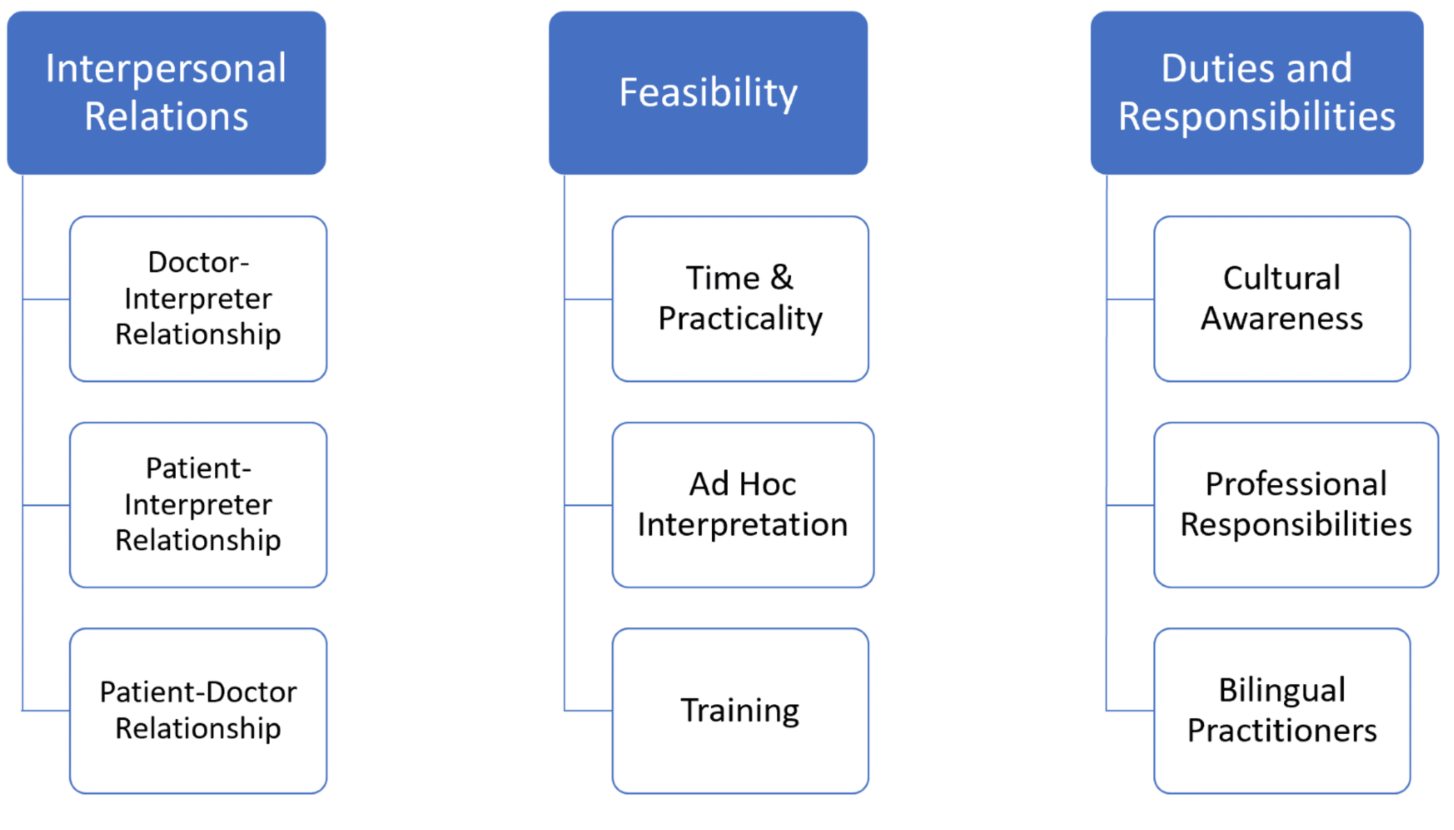
Thematic map of final themes.

Three significant themes surfaced from the focus groups: interpersonal relations, feasibility, and duties and responsibilities. These themes will be defined, discussed, and evidenced with data from the focus groups. The thematic map showing the three final themes is displayed in Figure 1.

Theme: interpersonal relations 

A theme that arose within the study was the varying experiences regarding the interpersonal relations of the members of the consultation. This theme can be defined as the contrasting and often conflicting three-way relationship that each participant of the consultation (i.e., the doctor, the interpreter, and the patient) had with one another.

The first interpersonal predicament that emerged in every focus group was the interprofessional relationship between the doctor and the interpreter. Medical students often noted a lot of doctors hesitated to fully trust their interpreter colleagues’ ability to translate precisely and proficiently. When discussing the challenges of working with an interpreter, Participant 2 touched on this mistrust when they remarked that they felt like interpreters condense important information from the doctor at will:

“Sometimes I don't feel like the interpreter translates everything, because I'm pretty sure, like the doctor would say a very long sentence in terms of treatment or scan results and the interpreter would just say a much shorter sentence. As if he summarised what the doctor was saying. So yes, I do think you do lose some information in the transfer, which is a challenge.”

Participant 2 believes that there may be potentially crucial elements of the patient investigation and management plan being omitted due to interpreter summarization. Excluding critical aspects from the patient can affect patient care and can ultimately be detrimental to the patient’s health. It may also affect the relationship between the two professionals during the consultation. This could be addressed, however, with some pre-consultation communication, as Participant 3 stated:

“I think they should have a briefing just before the consultation. You know, to just to give the interpreter a bit of the background of the patient, like what scan and why...just to make it a bit more practical, even when translating, if that makes sense. Especially if you are delivering sensitive or difficult news to the patient. Even saying to the interpreter, OK, I'm going to deliver some difficult news to the patient, so I don't want it to be like a shock to you when I tell this patient what their scan shows.”

Doctors should acknowledge that it may be distressing for the interpreter to hear certain information, and they may need a moment to process, it instead of learning it for the first time during the consultation. A pre-appointment briefing would aid the consultation immensely. The doctor and the interpreter would be managing and controlling the appointment in harmonious synchronicity as they would be following a plan that was created by each other prior to the arrival of the patient. This would benefit the entire relationship between the three members of the discussion, rather than a detached third party intruding on the consultation, as Participant 12 states:

“[On the team dynamic] it’s not great, but it doesn't help when the interpreter kind of doesn't know the background of the patient…it’s so impersonal, they come for the appointment and just literally leave.”

Participant 12 questions the indifferent nature of interpreters, implying that it squashes any hope of a professional relationship between the two experts, along with the patient. However, it can be argued that they may be pushed for time and have different appointments in different locations. However, this friction is corroborated by Participant 4:

“The doctors in the hospital really hate using the interpretation services, like they actually loathe it sometimes. It's really inefficient and it's difficult to grab a hold of a translator. So if you can find a doctor who knows the language, who's able to, you know, use his professional medical knowledge to put together the investigations, scans, and treatment, in one cohesive message as well as be able to provide, you know, the non-verbal communication too, I think that's always better than getting a translator, personally.”

This eagerness to cut out the extra step of incorporating interpreters, for the value of feeling more assured about explaining the results and the plan in the consultation, accentuates the degree of uncertainty or feelings of doubt experienced when using one. Not to mention the immense perceived practical benefits, which will be touched upon later. Participant 4 also indicated the importance of some of the nuances with regard to the transfer of emotion and non-verbal communication that doctors place an emphasis on, which some interpreters may fail to convey. 

Leading on from this, the interpreter-patient relationship is the next dynamic that emerged from the data. The students generally believed that interpreters needed to work on their interpersonal skills when transferring information across from doctor to patient. The students stressed the importance of non-verbal communication and spoke about how their medical training teaches them about the effective usage of displaying emotion and empathy. Whereas interpreters perhaps could draw some more attention to this, as Participant 9 comments:

“As a doctor or like the health care worker, you have to be a bit more aware like in terms of your facial cues and tone of voice. So being a bit more empathetic and so on, and so forth. But some of them are very cold. They just interpret without kind of any thought about facial expression, tone of voice or body language.”

This participant’s experiences highlight the significance of displaying the correct demeanor in the right context. Perfecting this skill can allow the clinicians and interpreters to form a trusting bond with the patient, which will in turn allow the patient to feel more at ease and heard, thus causing them to elicit more pertinent information. This is also touched upon by Participant 15 when identifying challenges in the consultation:

“One challenge is the transfer of the emotion behind the phrase. So I think what the patient sometimes has to do is to take the words that the interpreter said and remember the emotions that the doctor was using and try and like match the two together, because sometimes the interpreter, just literally blurts out a series of words, and the patient can find it hard to feel comfortable.”

Participant 15 also mentioned the significance of the phrasing of the words used in the translation. This can tie in with the doctor-interpreter relationship discussed earlier, perhaps if there was a clear plan formulated in a pre-appointment briefing, there may be more time for the interpreter to get into the right frame of mind emotionally and concentrate on the phrasing of certain terminology ahead of time, instead of focusing on simply verbatim interpreting. Participant 3 also speaks about this:

“The way they interpret it is very blunt interpretation…in the psychiatry ward they were interpreting to a patient [in a language I spoke], “you're a maniac.” And they were just very blunt instead of like saying to the patient, “You seem to have, you know, characteristics of a mania disorder.” 

Interpreting this way can potentially have detrimental effects on not only the patient-interpreter relationship but also the patient-doctor relationship. It is of critical importance to build a connection between healthcare staff and the patient, especially when they may be on the fringes of society such as those with completely different cultures and languages, who may struggle to integrate. Referring to improvements to patient care in this regard, Participant 7 retorts:

“Well, you could get the same translator as well just to build a rapport. I mean I’ve seen it happen elsewhere, not in my placements… I feel like it just breaks the ice a lot quicker like you don't need to go through the process of trust building.”

Participant 7 observed a simple yet highly effective tactic in their external employment that significantly enhanced the patient's relationship with healthcare staff and overall experience. The fact that almost all students had never seen this approach highlights weaknesses in the current system. Implementing a consistent healthcare team for patients, similar to the continuity of care provided in GP where patients value having a regular GP, could greatly improve the rapport between LEP patients and their doctors.

Following on from this, another interpersonal relationship aspect identified in the dataset was the patient-doctor relationship. Rapport building is central to the patient experience, and medical students seemed to feel that with an unsuitable or constantly changing interpreter, it would be exceptionally difficult to form an understanding with the patient. Participant 6 remarks:

“GP’s and patients, they build that connection over years right, so much so that they become almost on a first name basis and they treat generations of families. That may not be able to form if it's if there's like a random translator in between.”

The medical students acknowledged that it is a challenging skill on its own to build a rapport, it is one that is forged and perfected through years of experience, and factors such as this only make it more complicated. Participant 7 built on this and discussed the challenges of empathy and how it can feel like interpreters may impede this objective:

“I think it's hard to empathise and build a rapport with these patients…. I feel like to empathise you need to have a real sort of deeper connection with the patient, and when there is a translator I feel like there's this barrier.”

Participant 7 believes the mere presence of an interpreter acts as a “barrier” between the doctor and the patient, encumbering on the trust-building process. This psychological impediment of the medical students not being able to communicate directly with patients can also perhaps be coupled with the interpreter-doctor relationship issues as clearly there is a level of unease being displayed by the students in regard to having an interpreter present. However, the students do recognize that it would be almost impossible to conduct an appointment with these patients without interpreters, and that they are a crucial part of the multi-disciplinary team. As Participant 17 reiterates:

“I've seen a consultation done when there's clearly a language barrier but there's no translator available and you can't explain things about someone's health or recent blood tests or imaging results just through kind of hand gestures and pictures and things. It just doesn't get the message across the same at all…the patient was getting frustrated, she was struggling to breathe, she was uncomfortable anyway. But you could tell, she was getting frustrated purely from not being understood and not understanding fully what was being told to her.”

This participant has witnessed a truly ill-managed consultation wherein the physician deemed it appropriate to conduct the meeting without an interpreter and simply through drawings and actions. The patient was visibly irritated, and it seemed as though this approach was only successful in exacerbating her symptoms. Therefore, students acknowledge there is a definite need for interpreters. However, all the evidence above highlights the importance medical students place on interpersonal skills, both with members of the team and with the patient. This seems to be important to the medical students as it is something that they believe is vital to a productive meeting with a patient, to bring about an effective examination of the current problems, and to curate a meaningful management plan.

Theme: feasibility 

Feasibility concerns were another theme that was identified through coding the focus group data. This theme can be identified as the students’ trepidations surrounding the feasibility and viability of communicating with LEP patients. Feasibility concerns included time and practicality, ad hoc interpretive practices, and training issues.

Time and practicality were a topic that was recurrently brought up by the students. From their shadowing experience on placement, many of them observed that consultations involving an interpreter would take double or triple the regular consultation time. This would be even longer with the increasingly common phone interpretation services, as Participant 13 commented:

“I mean, it was like a 20, 30 minute GP consultation because the worst part is the delay, so when someone's talking via these translation services over the phone, there’s a weird, like, delay from the doctor speaking, then the translator doing their bit getting through to the person, and the person getting back to the translator, then them get back to you. So, like a conversation that would have taken like 30 seconds takes like three minutes on these services.”

Participant 19 also stated:

“You realise it takes a lot longer than you think. Like, I was on the phone for maybe half an hour just explaining stuff because sometimes there can be a mistranslation.”

In a fast-paced environment such as the medical sector, every second is crucial. In the UK, general practitioners spend an average of approximately nine minutes per appointment to be able to see and care for as many patients as possible throughout the day [17].

Whether or not this is ideal is a separate issue, but it is undeniably clear that appointments, where telephone interpretation is used, take a lot longer and may put a strain on the clinician and add unnecessary time pressure on the meeting. Participant 12 discussed the benefits and drawbacks of both in-person and phone interpretation services, and how doctors themselves realize that an in-person interpreter is a fortunate luxury to have in recent times:

“Because they've got the translator the doctors will go through, like everything because they think maybe the next time, they wouldn't have a translator. One of my consultants she like went through the entire like past medical history like double checked all the records and all that because they had an in-person translator. And I feel like doctors themselves know it works much better, but then I’ve also seen telephone consultations that, in comparison to this, like you can't build a rapport it's really awkward and you can’t elicit nearly as much information, but I guess it still works to achieve the main goal.”

There were mixed views on the viability of using telephone services, some students deeming it a hindrance that inhibits the use of non-verbal communication, and others viewing it as an undesirable, yet tolerable means to achieve the ultimate purpose of providing care. However, it seems that these telephone services will continue to become the new norm as medical students repeatedly view the accessibility of interpreters as one of the challenges hindering both the improvement of these consultations and more affectatious educational plans. Participant 2 points out:

“[On identification of any challenges] The availability of interpreters, I mean, if it's difficult to grab hold of an interpreter for the hospital staff, I imagine will be difficult to grasp them for teaching us in the medical school as well.” 

Building on this issue, the next quandary that arose from the focus groups was the use of ad hoc interpretive methods. The use of family members as interpreters is discouraged for a variety of reasons, one of the biggest being patient safety and well-being issues that are risked when allowing this practice to occur. As highlighted by Participants 11 and 18:

“I saw a patient in GP, she would keep on having these random miscarriages and the doctor would find random bruises on her body during the examination. She couldn't speak English at all, it would only be her husband speaking. So, the GP I think after her second miscarriage became quite concerned and, like tried to like get translator in but there were no translators available. So, she had to delay their appointment for by a couple of weeks to wait for a translator, so that was a big challenge…”

“A lady had just had a child and the midwife was coming over to visit and the husband was translating on her behalf. So, this lady didn't speak English, but the husband did. The husband was like mistranslating what she was saying to hide the abuse that was going on in the household.”

This menacing experience of mistranslation only becomes intensified when lack of interpreters is factored in, as patients will have to wait “weeks” in some cases for an appropriate interpreter. These safeguarding issues have huge implications in regard to patient safety, and even though the medical students are in fact specifically taught to not use family members as interpreters, doctors are all too lax and nonchalant about this practice. As accentuated by Participant 10:

“From my experience, like all the stuff that we were taught in Medical School - the whole “don't use family members”, I feel like in actual clinical practice it doesn't happen. There’s been so many times, where I've been in like sitting in GP appointments and after the consultation, I'm like “shouldn't you have had an interpreter, wouldn't that have been better” and everyone's just like “yeah I guess” but they don't actually get interpreters, even though it's like to us its so obvious that they need them because they're explaining really important stuff like needing X-Rays or CTs for red flag symptoms.”

From this, it shows that students themselves recognize it as an issue, and the doctors even agree with them; however, it is not something that is put into practice. Therefore, it is not an educational issue, rather a clinical or perhaps even a logistical problem, which would link back to the lack of interpreters available. This can potentially put patients at risk as understanding significant results such as CT scans or x-rays clearly can directly affect patient's adherence to the management plan. Additionally, one could argue that medical students will subconsciously pick these habits up from their superiors and continue treating patients in this manner.

Alternatively, if the family member is deemed trustworthy, doctors can rely too much on the relative and not incorporate the patient into the consultation. Leaving the patient as a mere bystander in their own appointment. This is illustrated by Participant 1:

“The patient’s daughter came, and it essentially turned into, you know, like the patient had a carer with them, like a patient who's got like, you know, cognitive impairment. And you just have the whole discussion with the carer… the actual patient every now and then would glance at me and smile and that was it. And he was just like completely out of it, like he wasn't really following it. So, I think that's one of the dangers of it, is that after a while, the doctor just becomes lazy.”

This type of carelessness from the doctor is harmful too, doctors must build a mutualistic relationship with their patients to achieve the highest levels of adherence and concordance. On the topic of improvements in this respect, one participant suggested the use of premade illustrations to aid the patient without the immediate use of an interpreter:

“People with disabilities and get those little cards with pictures on that they kind of can point to and be like “this”, surely they can make like a medical language version of that that caters to these patients.” 

This can be something that can be available on each ward in case of emergencies where there are no interpreters available, perhaps with images representing certain core symptoms or languages to help the healthcare staff identify what language interpreter needs to be booked.

The final and most substantial concern that was identified as part of the feasibility theme was issues surrounding training. Firstly, the medical students had quite a negative experience of their education on communicating with patients who do not speak English as their first language. Unanimously, the participants did not believe it to be sufficient and there was not one single student who believed they had adequate training to address these patients in the correct manner. Participant 3 stated:

“It was very brief and artificial, obviously, we didn’t really get the opportunity to practise.” 

Participant 5 also said:

“It's only just been observation at the moment, which in terms of reflection, I think isn’t ideal.” 

Participant 18 also mentioned:

“I don't think I feel confident enough to speak to a patient that doesn’t speak English.” 

Participant 18 reveals the lack of self-assurance a lot of students faced when posed with this question, all of them attributed it to the lack of exposure and practice to these kinds of patients. Understandably, some participants acknowledged the fact that even though their communication skills may be lacking on this front, they have limited time and exposure with patients anyhow. Therefore, any contact that they do have with patients must be prioritized in line with their core diagnostic competencies. Participant 14 states:

“If we ask nurses and doctors [for patients to practise on] we never get recommended patients that can't speak English and things like that. We're always signposted to patients that, you know are good communicators, like easy going and will talk to you. Rather than any sort of challenges, they try to protect us as much as they can.”

According to this participant, even the healthcare staff are unknowingly complicit in this. Perhaps combatting this issue should involve training the staff to allow the more junior students to see these good communicating patients, whereas the more senior students should be pointed toward the more difficult cases. As well as this, doctors who are more experienced in this field need to take some responsibility and inform and educate the students on the best ways to approach these situations, much like the consultant who Participant 11 was under:

“So, before the appointment, the doctor was like talking to me about the patient and she was telling me about how she approaches that situation. So, she said they had like impersonal translator coming in this time, so she was explaining to me like normally she brings in the translator first and like speaks to the translator and like briefs them. Then, she said she would call in the patient and she was telling me how she tries to speak directly to the patient, and like when she would ask the translators to go into like more detail, she was telling me how she would pick up on if they were like offering a direct translation or not.”

Consultants such as this one seem to be scarce as this was the only example of any teaching or even briefing that a doctor did. Justifiably then, the major view from the students with respect to improvements was simply to increase exposure and experience with LEP patients. Participant 20 commented:

“I think it would be useful if it was like a recurring thing each year. So, like, if you can introduce it first year and then slowly progress through the years rather than like a one hour lecture.”

Participant 15 also noted:

“More sessions like we said, like we've really only ever had one session with a translator like that's not really enough you know, the UK is so multicultural that actually we need more exposure to speak to people from different countries with different cultures, different language.”

Participant 15 raises an interesting argument, perhaps in medical schools based in larger, more diverse, and multicultural cities, they may place more education emphasis on LEP patient communication. Nevertheless, students who study in a certain university will not necessarily stay in the local area, therefore, this education must be universal and standardized. This is difficult, even for students to have similar experiences in the same university, as Participant 12 says:

“The limitations to like the improvements we said was that it's really difficult to make sure that every student has the same experience with language interpreters in a clinical setting because of the fact that we are all in different environments.”

In medical school, the scope of experience that students obtain is extremely broad. It would be very difficult to ensure the exact same experience for every student, especially when a university covers such a large geographical area and multiple trusts, such as Newcastle Medical School. Conceivably, more time with interpreters and their services would be beneficial in enhancing the experience of medical students. Having a basic understanding of their role and how they work will add insight and knowledge to an otherwise unfamiliar service, as Participant 14 expresses:

“We have placement days with a lot of other non-doctor specific roles, like patient support groups, to experience different aspects of patient care. So, I think a day with translators isn’t too much to ask for and could be beneficial to see how they would ideally work.”

This would benefit both parties as they would learn from each other and what the other requires for an ideal consultation scenario. In addition to building awareness and recognition of either party’s role, it also may in turn alleviate some of the tensions that the healthcare workers may have against interpreters.

The evidence of this section poses questions about the different aspects of the feasibility of the efficiency of time, practicality of the different methods of interpretation and the ethical use of ad hoc interpreters and whether they are being utilized and organized in the most efficiently and effectively.

Theme: duties and responsibilities 

The final significant theme that emerged from the participant's experience was duties and responsibilities. This theme can be characterized as the students’ experiences surrounding their diverging responsibilities within their specialized professional roles alongside their sense of duty. Medical students appeared to be conflicted in their sense of duty with their clinical role and responsibility. Students recognize that this group of patients does not get the same level of health management as English-speaking patients, in their opinion it is the language barrier that is a significant obstacle to overcome in terms of providing sufficient healthcare for all patients. Participant 13 admits:

“I do think that probably contributes to the slight difference in like health care given to different kinds of ethnicities, I do think part of it would be a language barrier, so it is definitely a problem that should be solved.”

However, they do concede that it is not simply a case of waiting for an interpreter, in some areas these services are stretched very thin and, in some instances, not available at the precise moment they may be needed, such as in cases of emergencies. As Participant 7 discusses:

“Its tough, especially when like in placement a woman came in with post-menstrual bleeding and taking a history from her was also hard. But the thing is, I had no choice because it was either the patient basically goes home having not had a consultation or she stays and has a poor consultation, because there's been no translator. But at least its better than nothing.”

Participant 4 also mentioned:

“I was put on the spot a bit by the doctor, but I translated anyway, ‘cos if it wasn’t for me, the consultation would have essentially not happened.”

The students’ moral sense of duty overrides their occupational responsibilities as they decide that getting at least some information from this patient for the doctor to look over is acceptable in this case. Rather than delaying the appointment and risking the patient being unable to recall the exact history and symptoms, or more dangerously, exacerbation of said symptoms and deterioration of health. The trainee doctors do feel a moral obligation to care for patients in any way they can, even if it means putting themselves in uncomfortable situations. Nonetheless, this can bring about occupational risks in that they may be doing more harm than good, Participant 11 touches on this subject:

“…There was a woman who was being given a C section and one of the reg’s like spoke the same language and they didn’t manage to get a translator, so he just came in and just translated for the whole operation. He was explaining everything, and he was like fluent so I mean that’s fine, but it was like three or four hours, out of like another reg’s time who would have had to cover for him on the ward.” 

This brings about an ethical healthcare priority dilemma for this registrar. Being a specialist registrar implies that this doctor was in a senior position, just below a consultant, therefore a lot of decisions for patient care lie on the shoulders of this doctor. Now, being the interpreter for this one patient may provide her with the ample healthcare that she needs, but being a senior doctor, he is leaving a whole ward’s worth of patients for this one patient, not to mention putting a lot of pressure on this other registrar by constraining him in absorbing his responsibilities alongside their own for a significant amount of time. Clearly, not the most efficient use of NHS resources. If this registrar isn’t performing any procedures, is just there as the interpreter, and is missing his usual obligations in the process, this decision of his may be up for judgment. Nonetheless, it being an emergency procedure such as a cesarean section does not help the situation as he would have had to make this decision in a split second.

Furthermore, the topic of bilingual clinicians was an issue that was brought up in every focus group. Most bilingual students expressed a willingness to use their other languages if necessity called for it, but they also acknowledged that there is certain specialist jargon that they would not know as they would not be used in everyday colloquial conversation:

“I spoke with her about her scan and obviously like words like colon or polyp. I was like, I haven't heard these words except like once in my lifetime in Arabic. So those words that were quite difficult. And you realise that unless you've been trained in it, it's actually quite a difficult thing to do.”

“It can be good at explaining it in the initial step, but if it's like quite like technical, you might need to still get a translator to explain the specific like specialist terminology from the imaging or biopsy results and complex procedures as most won’t know those words.”

Participants agreed that training in medical jargon is necessary to ensure quality and precise interpretation. Some suggested that medical schools should leverage their bilingual students by offering optional courses in these languages. This would enable students to accurately translate important imaging results and explain procedures. This could potentially be very beneficial, especially for the students who may want to expand their horizons or even the multi-lingual students who at some point may want to make use of their native language on patients from their nationality. Participant 5 remarked:

“Med schools could try to offer bilingual students training for their other language, like I don't understand how to use medical words in my own language, but I can speak it fluently apart from medical parts.”

Another participant also mentioned:

“I could like speak to the patient like take a brief history, but in terms of getting consent and actually giving them proper information about the operation or scan where they’d need to make an informed decision like I wouldn't be able to do that, I’d need to learn how to do that properly.”

Participant 10 raises an interesting point on the issue of consent. When providing and obtaining informed consent, the doctor must provide all the information necessary, with no ambiguity in communication especially when using specialist medical jargon. The clinician needs to be assured that the patient has been given all the information needed and they have absorbed and understood this information before making their decision. Using a non-professional interpreter can raise ethical concerns as these risks uncertain transfer and understanding of the information. This is clearly reflected in the anxieties of the participants.

Additionally, cultural awareness was another issue that emerged from most of the focus groups data. The bilingual students strongly believed that cultural awareness education was something that was severely lacking in their teachings from medical school. This will then be reflected in the way they treat both patients and colleagues when they become doctors. An example of this is shown in Participant 17:

“Well, in the paediatrics A&E they found out that the patient was Chinese. The nurses asked if I was Chinese, which I am, but I speak Mandarin, the patient spoke Cantonese and my Cantonese is so limited. I think the patient got away with broken English, but like it was not a situation I was comfortable with at all, in terms of explaining a whole medical history, it was not ideal.”

The pressure placed on this student by the healthcare staff illustrates the lack of cultural awareness of the situation. Just by presuming that because the student was Chinese, they thought that they would understand each other. This shows that there is a need for educating the healthcare sector as the UK is a multicultural region and it should be commemorated by teaching its working population about the various cultures that they may come across with. In light of this, certain cultural practices may be inappropriately construed by some people who do not understand these traditions, as Participant 16 discusses:

“Most likely if a patient doesn't speak English, the chances of them being from a different culture is really high, and I think if you don't have that cultural awareness, you're not going to be able to pick up on things like safeguarding. So, something that someone might think it's really weird could be normal for that group of people, so I think just having more diversity and having more examples and scenarios in our education, just introducing more like cultural things so that people are more aware of them.”

Participant 6, among other participants, calls for more diversity in teaching and training for medical students. Especially regarding larger populations of cultures in the UK and their practices that may directly involve the healthcare sector. If medical schools endeavored to include more diverse scenarios for their students to use and learn from, this could go a long way toward providing this foundational cultural understanding that they may need, which they will hopefully build upon as their career progresses. Even appointments that may revolve around what some cultures may regard as taboo topics would greatly benefit from even a slight cultural consciousness to organize gender-matched interpreters, as Participant 3 describes:

“If you're speaking about something that’s seen as sensitive in certain cultures, I'm not sure if there's a preference, but you should be able to pick and choose gender when you're picking a translator. It was so awkward just because it was a male translating. If it was a female, I think it would just be a lot smoother and it would have been a lot easier to translate.”

Students who came from Black And Minority Ethnic (BAME) backgrounds also believed that due to their upbringing, they had an increased empathy for patients from non-English backgrounds and were therefore able to view these patients in a more holistic manner, thus treating the patient rather than the problem:

“It's because we're from a BAME background that we relate to this issue of cultural differences with like language barriers and stuff. But to other people it's like they see a patient that doesn’t speak English and they think that's the only problem, and it's like if you translate it the problem is solved and it's not like that, it's actually a lot, a lot bigger than that.”

Having an understanding of cultural differences, along with the conflicts and difficulties that arise with this insight, allowed these BAME students to express their thoughts and emotions about this topic in a deeper manner. A lot of them sharing anecdotes about family members having difficulties accessing the NHS due to language and cultural barriers, and even some of them being ad hoc interpreters for these family members as they were considered the “medical experts” who would be able to relay the information more effectively and efficiently.

The results above showcase that distinctive cultural barriers have become obstacles in the perspective of medical students and are distorting their views on the boundaries of their moral sense of duty and their work-related responsibilities.

## Discussion

This study aimed to assess the medical student experience regarding communicating with LEP patients. Overall, three crucial themes surfaced in students’ experiences. Interpersonal relations, feasibility, and duties and responsibilities. These findings will be conceptually discussed with regard to preceding literature and established phenomena. Methodological weaknesses and implications for future training will also be reviewed.

Interpersonal relations

Interpersonal relations were a key theme that emerged from the investigation, involving the differing relationships among participants in the consultation. Medical students noted that both they and their supervising practitioners found it difficult to fully trust interpreters and the precision of their interpretation, especially for significant investigations such as imaging results. These findings reflect a systematic review conducted by Brisset et al. [18]. In this meta-ethnography, researchers investigated 66 qualitative studies on interpreting in the healthcare sector and concluded that trust and control issues arise between patients, interpreters, and doctors. Results found that many interpreters noted doctors expressing distrust in their competence to interpret satisfactorily.

Similar to the results of this present study, Brisset et al. found that clinicians believed the presence of an interpreter could cause a perceived loss of intimacy with the patient [18]. The findings of the current study also alluded to the mismanagement of control as a cause of friction between the two professionals. Brisset et al. also determined that doctors may be concerned that the interpreter could influence their rapport with patients or that the patient could become passive and inactive in the consultation, relying on the interpreter, much like the examples provided by the students with relatives as ad-hoc interpreters [18]. The authors also highlighted that despite being required to be passive and neutral in the consultation, interpreters are expected to deliver emotional assistance and be cultural intermediaries. These many responsibilities can understandably be difficult to balance, especially when considering each patient's unique needs and the varying degrees of each role required.

With this in mind, Brisset et al. reviewed the patient-interpreter relationship and established that while patients have confidence in qualified interpreters' expertise, they may favor relative interpreters due to established relationships, adding that too much emotional proximity with an interpreter is not valued [18]. Another paper illustrated that patients' relationships with interpreters may not be solely based on linguistic capabilities but also on birthplace, faith, dialect, sex, and more [19]. This difficult balancing act may explain why participants identified many examples of interpreters lacking soft skills and appropriate bedside manners.

The systematic review paper exemplifies the dynamics and complexities of the three-way consultation and the herculean effort of managing control and balance within it. If one of the protagonists' challenges for control in a misguided, miscalculated manner, the balance could be disturbed, affecting the quality of relationships. Any trust that had been developing may fade, adversely affecting the quality of care and resulting in poor outcomes for the patient.

The results revealed that participants experienced feasibility trepidations, such as time and practicality, ad hoc interpretation, and training concerns. Studies show that these views are shared among their seniors, with potential obstacles to quality interpretation being perceived time and practicality issues related to availability and communication with a professional interpreter and dependence on unqualified ad hoc interpreters [20]. Utilizing patient’s relatives, acquaintances, or even untrained staff as interpreters is undoubtedly helpful, but its effectiveness is restricted by the interpreter's familiarity with medical vocabulary [20]. Ramirez et al. discuss that doctors continue to rely on non-professional interpreting methods, with clinicians and nurses interpreting 49% of the time, other staff 27%, and relatives and friends 12% of the time [20]. This data corroborates the experiences of the students in this current study, although most doctors knew that using ad hoc interpreters was not ideal, for the sake of feasibility, it was an overlooked misdemeanor. Indeed, Flores et al. discovered that ad hoc interpreters were correlated with nearly twice the likelihood of committing errors of possible clinical significance, compared to professional interpreters, which is anything from history to blood tests, to imaging reports [21]. A separate study also concluded that imprecise interpretation transpired twice the rate in ad hoc interpretation compared to professional interpretation [22]. Considering the literature, the nonchalant behavior the students have observed surrounding the use of ad hoc interpreters could be seen as potentially negligent. Ad hoc interpreter errors are well established to jeopardize patient well-being and are linked with avoidable harm and serious injuries; therefore, clinicians can be seen as significantly decreasing their quality of care.

In terms of training concerns, research aligns with the present study's students, indicating that many medical schools offer minimal focus on effective interpreter collaboration education [23]. Furthermore, potentially due to this lack of education, the consensus suggests students feel woefully unprepared to care for LEP patients, a sentiment shared by the present study's participants [24]. If students find working with interpreters frustrating, this will likely impact how they care for potential LEP patients, potentially making it challenging and compromising the quality of care provided. Encouragingly, increased training, whether delivered through traditional lecture-based courses [25] or even online e-learning modules [12], has been shown to improve ease with interpreters, understanding of interpreter responsibilities, and awareness of vocabularies used by patients from diverse backgrounds [26,27].

Duties and responsibilities

Duties and responsibilities emerged as a major theme in the students' experiences, particularly regarding cultural awareness and the prospect of being bilingual practitioners. The students felt their curriculum and educators displayed a lack of cultural conscientiousness, hindering their ability to empathize with the LEP population. Rodriguez et al. noted similar findings in 2011, with Hispanic medical students feeling more equipped to care for LEP patients, substantiating the view that an imperative tactic to decrease racial health inequalities is to proliferate the diversity of the healthcare labor force [24].

The results from this study also displayed mixed emotions regarding the use of students' bilingual abilities. Some students were put in uncomfortable situations to interpret and would often lean on their moral sense of duty toward the patients. These findings emphasize the tension bilingual students encounter when treating patients in their second language and accentuate the necessity for guidance in this area. One survey also demonstrated that a significant percentage of bilingual pupils felt uncomfortable, pressured, apprehensive, uneasy, and uncertain about interpreting in their other language [28].

Whether or not students believe it is a good idea, relying on staff for their bilingual capabilities can be careless use of time, especially when that staff member could be doing more meaningful tasks, as one of the students in this study mentioned. With this in mind, another survey uncovered that 58% of bilingual physicians in one hospital were summoned to interpret almost daily [20]. These physicians spent over two hours a week interpreting for other staff members. This indicates that utilizing clinicians for interpreting may not be the most clinically optimal or cost-efficient strategy.

Limitations 

One noteworthy limitation of the study was that because of the COVID-19 restrictions, focus groups were held entirely online via Zoom rather than face-to-face. Although they overcame the geographical barriers of qualitative research, there are caveats. In a critical comparison study comparing traditional face-to-face focus groups to online focus groups, the study panel of experts unanimously agreed that the results from the face-to-face focus groups were the most insightful and encompassed the most features of the research question [29]. The experts went on to distinguish the findings attained from the face-to-face focus groups as personal, spontaneous, relevant, and well-argued, whereas they classified the answers from the online group as spontaneous but superficial [29]. Generally, the literature indicates that the findings may have been richer if the focus groups had been conducted face-to-face.

Another drawback is the probability of volunteer bias. Firstly, because online groups necessitate access to and capability with modern technology and the internet, engagement with the study was therefore restricted to individuals with proficiency operating Zoom. Additionally, the sample size being only 20 may be primarily due to the impact of COVID-19 and the natural tendency of students to prioritize their studies and personal lives. However, the study participants largely fit the general cultural study population of the area as all languages that the participants spoke were in the top 10 most spoken languages of the area. Furthermore, because snowball sampling was employed, participants were self-selected. Making this commitment to the study could mean the participants have a vested interest, hence the thoughts and feelings expressed by them may not represent those who do not have a personal interest in the topic. Nevertheless, as thematic analysis explores correlations within the dataset completely rather than from particular persons, this weakness is not an alarming one.

Future implications 

This study has highlighted multiple suggestions for future training and practice. The most apparent implementation is more consistent teaching and increased exposure. Starting from the first year with role play and theory, followed by more focused teaching as the students progressed would ensure maximum preparation. Supervisors must not be hesitant in assigning senior students more complex cases. Learning more about interpreters and their role, the benefits, and drawbacks of the different kinds of interpreters, and how to effectively work with them. Figures propose that teaching medical students how to successfully collaborate with interpreters may produce greater preparedness to care for LEP patients [24]. For doctors, implementing a briefing period preceding the appointment, and reviewing any objectives and strategies, may aid in creating a considerate, accommodating, and efficient working environment. Cultural awareness education is essential as it has been shown that clinicians who received this teaching considered themselves more able to treat a more diverse population [24]. Efficacious guidance is vital to improve the multi-disciplinary team, enhance patient care, and decrease health disparities for LEP patients. Lastly, there arises a prospect of embedding a standardized functionality in NHS electronic health records. This feature would enable the instantaneous translation of clinical notes, and investigations such as scan or procedure results. Presenting a valuable avenue for enhancing patient outcomes in the dynamic landscape of contemporary healthcare.

## Conclusions

The present study observed that the students experienced difficulties concerning interpersonal relations, feasibility, and duties and responsibilities regarding caring for LEP patients. Experiences encountered by these participants should be applied to modify teaching, as educational interventions must focus on genuine experiences and applicability. Education in improving collaboration between professionals and cultivating trust and teamwork within a multi-disciplinary team has been established as an indicator for improved health outcomes for patients. Practical exposure-based adjustments encouraging the usage of professional interpreters, teaching staff, and students to cooperate with interpreters successfully could significantly enhance patient outcomes. Additionally, in this current era marked by the NHS's transition into adapting to modern times, it is proposed that incorporating a standardized feature for real-time translation of clinical notes, imaging results, and biopsy findings within these systems will likely facilitate the decrease in health inequalities for the LEP population.

## Appendices

**Table 2:** Appendix 1 - invitation letter

Invitation letter
Dear Student/colleague, We are writing to invite you to participate in a research project by taking part in a focus group. Title: Treating patients with limited English proficiency: Medical student’s experience in teaching and practice The study is interested in researching the personal experiences of medical students with regards to both their teaching and actual practical encounters communicating with patients who have a limited English speaking proficiency. The aims of this study are to investigate what you as a student thought of your training and preparation for engaging with non-English speaking patients, exploring any experiences with non-English proficient patients during your training or whilst out on placement, any challenges you have encountered, and to hear your thoughts on any future prospects for education on this topic. A Participants Information sheet is enclosed to provide further information of the study, and what would be required from you if you kindly choose to take part. A copy of the consent form is also provided. Please note that this is just for you to look over and doesn’t need to be completed until the day of the focus group. A focus group has been arranged for …... If you are interested in taking part in the study please contact me, [EMAIL HAS BEEN REDACTED FOR PRIVACY PURPOSES] If you have any further questions, please do not hesitate to contact the research team. Thank you for your time, With Kind Regards, Mehrab Durrani, Newcastle University.

**Table 3:** Appendix 2 - consent form

Consent form
Consent Form for Medical Student Focus Group Participants Title of Study: Treating patients with limited English proficiency: Medical student’s experience in teaching and practice. Thank you for your interest in taking part in this research. Please complete this form after you have read the Information Sheet and/or listened to an explanation about the research study. You will be given a copy of this Consent Form. Please initial box to confirm consent 1. I confirm that I have read the information sheet dated 30/03/2021 for the above study, I have had the opportunity to consider the information, ask questions and I have had any questions answered satisfactorily. ___ 2. I understand that my participation is voluntary and that I am free to withdraw at any time without giving any reason. I understand that if I decide to withdraw, any data that I have provided up to that point will be omitted. ___ 3. I consent to the processing of my personal information (name, video recorded image, audio recording of your voice) for the purposes of this research study, as described in the information sheet dated 30/03/2021. ___ 4. I understand that my research data may be published as a report. ___ 5. I understand that my research data may be looked at by individuals from Newcastle University where it is relevant to my taking part in this research. ___ 6. I consent to being audio and video recorded and understand that the recordings will be destroyed immediately after transcription and stored anonymously on password-protected software and used for research purposes only. ___ 7. I agree to take part in this research project. ___ Participant: Name of participant: Signature: Date: Researcher: Name of researcher: Signature: Date:

**Table 4:** Appendix 3 - information sheet

Information sheet
Project title: Treating patients with limited English proficiency: Medical student’s experience in teaching and practice. Invitation and Brief Summary You are being invited to take part in a research study. The research is being conducted by Mehrab Durrani, within the School of Psychology, Newcastle University. Before you decide whether or not you wish to take part it is important that you understand why the research is being done and what it will involve. Please read this information carefully and discuss it with others if you wish. Take time to decide whether or not you wish to take part. If you do decide to take part, you will be asked to sign a consent form. However, you are free to withdraw at any time, without giving any reason and without any penalty or loss of benefits. What is the purpose of the research? The study is interested in researching the personal experiences of medical students with regards to both their teaching and actual practical encounters communicating with patients who have a limited English speaking proficiency. The aims of this study are to investigate what you as a student thought of your training and preparation for engaging with non-English speaking patients, exploring any experiences with non-English proficient patients during your training or whilst out on placement, any challenges you have encountered, and to hear your thoughts on any future prospects for education on this topic. What does taking part involve? Should you choose to take part in this research study, you will take part in an online group focus study, involving approximately 4 to 6 other medical students at your stage of your medical training, via Zoom. The focus group will follow a semi-structured interview form which means that the interviewer will have a series of open ended topics to cover but there is freedom to allow the conversation to follow a natural development. Topics that will be covered will involve discussing current training, thoughts and feelings about present education, examples of experiences and challenges interacting with patients with limited English proficiency, experiences and challenges working with interpreters, whether or not you think enough is being done and if not, what else needs to be done to help you enhance your communication skills with these patients. It is estimated that the focus groups will last around 45 minutes to 1 hour, but they will be flexible enough to last longer if needs be. What information will be collected and who will have access to the information collected? The information collected from the focus group responses will be used in the completion of Mehrab Durrani's Clinical Foundations of Psychology dissertation research project. The results of the focus group will be submitted in the form of a written report. If suitable, the results may be published in an academic journal and presented at a research conference. At all times, your identity will remain anonymous. We will use your name and contact details (email address, telephone number) to contact you about the research study. We will generate a unique 5 digit code to allow for anonymity whilst analysing the results of the focus group. Individuals at Newcastle University may look at your research data to check the accuracy of the research study. The only individuals at Newcastle University who will have access to information that identifies you will be individuals who need to contact you for any pertaining reasons or audit the data collection process. As the focus groups will takes place over Zoom, it will be transcribed through the platform and then subsequently deleted from their dedicated storage and the transcripts will be anonymised and stored in a password protected software. Why have I been invited to take part? In order to investigate this, we are asking medical students from all stages of their university education, who are fluent in spoken English, to take part in an online focus group study. What are the possible benefits of taking part? There are no direct benefits from participation in this study. However, your participation will help to increase knowledge in the topic area and could inform future research regarding how best to teach and communicate with patients who are not proficient in English. What are the possible disadvantages and risks of taking part? The researchers do not anticipate any negative effects or risks of taking part in this study. The focus group includes questions which ask about your experiences regarding teaching and approaching non-English speaking patients. These questions are not of a personal nature and are not expected to cause distress. If you feel as though this may cause you distress, it is advised that you do not take part. Has this study received ethical approval? This study has received ethical approval from the Research, Policy, Intelligence and Ethics Team, Newcastle University Research Office, on 18/03/2021. Who should I contact if I have any concerns about the research? If you have any questions for the researchers or wish to raise any concerns, please contact the researchers at the details below. Should you have any complaints concerning the manner in which this research is conducted, please contact the Chair of the Faculty Ethics Committee at fms.ethics@ncl.ac.uk. If you wish to raise a complaint on how your personal data is handled, you can contact the School of Psychology manager Lorna Nelson; email: Lorna.Nelson@newcastle.ac.uk If you are not satisfied with their response you can complain to the Information Commissioner’s Office (ICO): https://ico.org.uk/ If for any reason you are concerned regarding any of the issues raised whilst completing this focus group, please feel free to discuss these matters with your personal medical tutor. Who should I contact for further information relating to the research? If you have any questions, please do not hesitate to contact the research team using the following details below: [EMAIL REDACTED FOR PRIVACY REASONS] School of Psychology, Margaret Barbour Building, Wallace Street, Newcastle upon Tyne, NE2 4DR Thank you for considering taking part in the research study. If you would like to participate in this study please get in touch with me with the email above. Regards, [MY NAME] [SUPERVISORS NAME]

**Table 5:** Appendix 4 - interview aims, schedules, and prompts

Interview aims, schedules, and prompts
Aims: To explore experiences students had with non-English speaking patients. Identify whether students think they are being prepared enough to treat non-English speaking patients? Identify training needs that would better prepare students to communicate and handle such cases - get suggestions from them Interview Schedule - See prompts in red Ascertain current teaching and training throughout medical school in relation to the topic. Thoughts and feeling surrounding current training. Experience of working with non-English speaking patients In relation to patients In relation to other professionals (other members of healthcare staff, interpreters, ad hoc interpretation usage such as other members of staff/themselves if bilingual) Dynamic of the team Identification of any challenges How these were dealt with How would these be dealt with Challenges personally experienced, or heard about from someone else (colleague/student) Identify what skills they think is required to be able to communicate effectively. Do they think they are getting adequate training to gain these skills? Possible improvements/suggestions for teaching and training. Why these improvements would be effective Any obstacles in implementation Effect these would have/ difference they would make Potential training with interpreters/interpretation students?

**Table 6:** Appendix 5 - debrief sheet

Debrief sheet
Thank you for taking time to participate in this survey. What was the purpose of this study? The study is interested in researching the personal experiences of medical students with regards to both their teaching and actual practical encounters communicating with patients who have a limited English-speaking proficiency. The aims of this study are to investigate what you as a student thought of your training and preparation for engaging with non-English speaking patients, exploring any experiences with non-English proficient patients during your training or whilst out on placement, any challenges you have encountered, and to hear your thoughts on any future prospects for education on this topic. What are the expectations of the study? The results of the study will help to contribute to our understanding of the teaching surrounding communicating with non-English speaking patients and whether it is sufficient or needs updating. The results will therefore increase knowledge in this topic area and could inform future research regarding how best to support patients who can not speak English proficiently. Do I still have the right to withdraw? Yes. Please contact the researchers via email, with your name and approximate time that you completed the focus group, within 2 weeks of completion of the survey if you wish to withdraw your data. Issues raised by the research. As noted on the participant information sheet, if for any reason you are concerned regarding any of the issues raised whilst completing this focus group, please feel free to discuss any matters with your personal medical tutor. Where can I learn about the study’s findings and what if I have any further questions about the research? If you wish to find out the study’s findings, once the study is complete, or you wish to ask any further questions about the research, you can do so by contacting the researchers via email.at the following details: [EMAIL REDACTED FOR PRIVACY] [SUPERVISOR EMAIL REDACTED FOR PRIVACY] Thank you once again for your time and participation.
